# A Short-Term High-Fat Diet Alters Glutathione Levels and IL-6 Gene Expression in Oxidative Skeletal Muscles of Young Rats

**DOI:** 10.3389/fphys.2019.00372

**Published:** 2019-04-10

**Authors:** David E. Andrich, Lilya Melbouci, Ya Ou, Nickolas Auclair, Jocelyne Mercier, Jean-Christophe Grenier, Fábio Santos Lira, Luis B. Barreiro, Gawiyou Danialou, Alain-Steve Comtois, Jean-Claude Lavoie, David H. St-Pierre

**Affiliations:** ^1^ Département des Sciences de l’Activité Physique, Université du Québec à Montréal (UQAM), Montréal, QC, Canada; ^2^ Groupe de Recherche en Activité Physique Adaptée (GRAPA), Université du Québec à Montréal (UQAM), Montréal, QC, Canada; ^3^ Département des Sciences Biologiques, Université du Québec à Montréal (UQAM), Montréal, QC, Canada; ^4^ Centre de Recherche du CHU Sainte-Justine, Montréal, QC, Canada; ^5^ Department of Physical Education, São Paulo State University, São Paulo, Brazil; ^6^ Département de Pédiatrie, Faculté de Médecine, Université de Montréal, Montréal, QC, Canada; ^7^ Royal Military College Saint-Jean, Saint-Jean-sur-Richelieu, QC, Canada; ^8^ Département de Nutrition, Faculté de Médecine, Université de Montréal, Montréal, QC, Canada

**Keywords:** high-fat diet, young rats, muscle glutathione, oxidative stress, inflammation, gene expression

## Abstract

Obesity and ensuing disorders are increasingly prevalent worldwide. High-fat diets (HFD) and diet-induced obesity have been shown to induce oxidative stress and inflammation while altering metabolic homeostasis in many organs, including the skeletal muscle. We previously observed that 14 days of HFD impairs contractile functions of the soleus (SOL) oxidative skeletal muscle. However, the mechanisms underlying these effects are not clarified. In order to determine the effects of a short-term HFD on skeletal muscle glutathione metabolism, young male Wistar rats (100–125 g) were fed HFD or a regular chow diet (RCD) for 14 days. Reduced (GSH) and disulfide (GSSG) glutathione levels were measured in the SOL. The expression of genes involved in the regulation of glutathione metabolism, oxidative stress, antioxidant defense and inflammation were measured by RNA-Seq. We observed a significant 25% decrease of GSH levels in the SOL muscle. Levels of GSSG and the GSH:GSSG ratio were similar in both groups. Further, we observed a 4.5 fold increase in the expression of pro-inflammatory cytokine interleukin 6 (IL-6) but not of other cytokines or markers of inflammation and oxidative stress. We hereby demonstrate that a short-term HFD significantly lowers SOL muscle GSH levels. This effect could be mediated through the increased expression of IL-6. Further, the skeletal muscle antioxidant defense could be impaired under cellular stress. We surmise that these early alterations could contribute to HFD-induced insulin resistance observed in longer protocols.

## Introduction

Obesity has become a major health, social, and economic burden worldwide (Hruby and Hu, 2015). This is particularly concerning among children, since the prevalence of obesity and overweightness in this age group has risen by nearly 10% in the last 4 decades (Rao et al., 2016). Indeed, the risk of carrying excess weight into adulthood and of developing morbid obesity is much greater among obese children and adolescents (The et al., 2010). Overweight children are also more at risk of developing obesity related diseases like type 2 diabetes and the metabolic syndrome in later stages of life (Biro and Wien, 2010).

A sedentary lifestyle and poor diet quality are known as two main contributors to obesity, as calorie-rich diets promote a positive energy balance leading to weight gain. Obesity increases the risk of developing a large number of metabolic disorders (Head, 2015). For instance, we have recently reported that only 2 weeks of high fat diet (HFD) significantly altered contractile functions of the oxidative skeletal muscle soleus in young rats, although the same result could not be observed in the glycolytic *extensor digitorum longus* (EDL) muscle (Andrich et al., 2018b). Hence, long known to cause excess lipid accumulation (Peckham et al., 1962), HFDs have also been shown to stimulate the production of reactive oxygen species (ROS), thus leading to oxidative stress (Auberval et al., 2014). Strong evidence shows that sub-clinical inflammation and oxidative stress are two of the main contributors for the pathogenesis of metabolic dysfunctions in the obese state (Galassetti, 2012). It appears as though HFD-stimulated excess ROS production (Vial et al., 2011) can precede observable weight gain and insulin resistance (Matsuzawa-Nagata et al., 2008), indicating that oxidative stress might be a result of the diet itself and not a consequence of excess lipid accumulation. Further, HFD induces inflammation and oxidative stress in the skeletal muscle of rodents (Yokota et al., 2009; Gortan Cappellari et al., 2016). Beyond its role in locomotion and posture maintenance, skeletal muscle is a key player in the regulation of metabolic homeostasis (Frontera and Ochala, 2015). In fact, skeletal muscle insulin resistance is considered as the primary cause of type 2 diabetes (DeFronzo and Tripathy, 2009). Skeletal muscle dysfunctions can be induced by oxidative stress in type 2 diabetes patients (Tsutsui et al., 2011; Diaz-Morales et al., 2016; Wang et al., 2016), highlighting its role in the pathogenesis of the disease (Giacco and Brownlee, 2010). Further, 6 weeks of HFD has been shown to decrease reduced glutathione (GSH) levels, an important antioxidant, in the gastrocnemius of 8-week-old Sprague–Dawley rats (Anderson et al., 2009). After a similar exposure to HFD, higher glutathione disulfide (GSSG) levels and a lower GSH:GSSG ratio were also observed in the gastrocnemius muscle of 18-week old rats (Fisher-Wellman et al., 2013). However, the early mechanisms underlying such alterations remain to be elucidated. Further, it was previously shown that glutathione levels are more prone to undergo HFD-induced alterations in oxidative muscle (Pinho et al., 2017). Therefore, this study aimed to investigate the effects of a short-term (14 days) HFD on glutathione metabolism in the soleus muscle of young rats. To do so, we measured glutathione levels as well as gene expression of known factors regulating glutathione metabolism, oxidative stress, and inflammation. Thus, we hypothesized that a short-term exposure to an obesogenic diet alters glutathione production and redox potential while inducing oxidative stress and inflammation in the soleus muscle.

## Materials and Methods

### Animal Procedures

This study was carried out in strict accordance with recommendations of the National Institutes of Health guide for the care and use of Laboratory animals. Before undergoing the experimental work, the protocol was approved by the *Comité Institutionnel de Protection des Animaux* (CIPA) of UQAM (Permit Number: 0515-R3–759-0516). After a 3-day acclimatization period at UQAM’s animal facility, young (100–125 g; approximately 4 weeks old) male Wistar rats (Charles River, St-Constant, QC, Canada) were randomly fed with a regular chow diet (RCD; *n* = 13) or HFD (*n* = 12) for 14 days and submitted to a 12-h light/dark cycle starting at 06:00. Animals were given *ad libitum* access to the diets and water throughout the experimental protocol. Sacrifice was achieved under anesthesia (3% isoflurane at 0.5 L/min of O_2_) after a 4 h fast to standardize the feeding status of each animal. The soleus (SOL) muscle of both legs was collected for glutathione determination and RNA extraction.

### Diets

Physiological fuel values were calculated from modified Atwater factors (3.5 kcal/g carbohydrate; 3.5 kcal/g protein; 8.5 kcal/g fat). The high fat diet was prepared from purified food-grade reagents according to a commercial formulation (D12492 diet, Research Diets Inc., New Brunswick, NJ, USA). It had a macronutrient weight content of 26.3% carbohydrate (19.2% kcal), 26.2% protein (19% kcal), and 34.9% fat (61.8% kcal) and a physiological fuel value of 4.80 kcal/g. Carbohydrate sources were maltodextrin and sucrose (64.5 and 35.5%, respectively), protein sources were casein and L-cystine (98.5 and 1.5%, respectively), while lipid sources were lard and soybean oil (90.7 and 9.3%, respectively). The diet also contained cellulose (64.6 g/kg), calcium carbonate (7.1 g/kg), dicalcium phosphate (16.8 g/kg), potassium citrate (21.3 g/kg), and choline bitartrate (2.6 g/kg), as well as mineral (12.9 g/kg) and vitamin (12.9 g/kg) mixes. The regular chow diet (Charles River Rodent Diet # 5075, Cargill Animal Nutrition, MN, USA) had a macronutrient weight content of 55.2% carbohydrate (65.6% kcal), 18% protein (21.4% kcal), and 4.5% fat (13% kcal) and a physiological fuel value of 2.89 kcal/g.

### Glutathione Measurements

Immediately after collection, 0.25 g of SOL muscle was homogenized (2 × 10 s with Polytron Teador; Biospec Products Inc, Dremel-Racine, WI) in 1.25 ml of iced and freshly prepared 5% (w/v) metaphosphoric acid (Fisher A280–100) and centrifuged for 3 min at 7200 *g*. Pellets and supernatants were kept at −80°C until protein and glutathione determinations, respectively. Reduced glutathione (GSH) and glutathione disulfide (GSSG) were quantified by capillary (75-μm × 50-cm silica) electrophoresis (75 mM boric acid and 25 mM Bis-Tris, pH 8.4, 28°C, 18 kV) as described previously (Lavoie et al., 2008). The redox potential was defined as the half-cell reduction potential of the GSSG (2H^+^/2GSH couple) and calculated by using the Nernst equation (25°C, pH 7.0) (Turcot et al., 2009).

### RNA Extraction

Collected tissue samples were stored in RNAlater stabilization solution (Ambion) and stored at −20°C for later use. About 15 to 60 mg of tissue per sample was homogenized in 1 ml of TRIzol Reagent (Ambion) with a TissueLyserII homogenizer (Qiagen) and extracted according to the manufacturer’s instructions. Samples were further processed using the PureLink RNA Mini Kit (Ambion), and contaminating DNA was removed *via* DNase on-column digestion. A BioDrop spectrophotometer was used to determine RNA concentrations, and the ratio of absorbance at 260 and 280 nm is used to assess purity. RNA integrity was evaluated by visualization of intact 18S and 28S RNA bands following agarose gel electrophoresis. SuperScript VILO Master Mix (Invitrogen) was used to synthesize cDNA with 1 μg of RNA per 20 μl reaction.

### RNA Sequencing

RNA sequencing methodology was adapted from Pai et al. (2016). Briefly, libraries were prepared using the Illumina TruSeq protocol. Once prepared, indexed cDNA libraries were pooled (six libraries per pool) in equimolar amounts, and the majority was sequenced with single-end 101 bp reads on the Illumina HiSeq4000. Low quality score bases and adaptor sequences were first trimmed using Trim Galore (version 0.2.7). The resulting reads were then mapped to a genome reference sequence (Ensembl Rnor_6.0 release 81) with STAR (version 2.4.2) using the 1-pass protocol. The number of mismatches allowed for the pairs was of 5, and a soft-clipping step that optimizes alignment scores was automatically applied by the STAR software. Read counting on each gene was done with HTseq (version 0.6.1p1), which was launched separately on each alignment file with the intersection-nonempty option, supported by SAMtools (version 0.1.19) using the same gene reference file as for the alignments.

### Statistical Analyses

Sample sizes were calculated as recommended (Charan and Kantharia, 2013) using data from previously published studies, as well as our own pilot studies using power set at 0.8 (80%) and significance set at *p* < 0.05. All values are presented as means ± SD, except where noted. Normality was assessed using the Shapiro–Wilk test. Unpaired Student’s *t* tests were used to compare values between the two groups. Statistical analyses were performed using the SPSS 16.0 (IBM Corporation, Armonk, NY) software. For RNA-Seq analyses, the DESeq2 (version 1.18.1) software was used to identify genes with a significantly different expression in the HF group. A FDR-adjusted *p* < 0.10, corresponding to the treatment variable, and an absolute fold change of mean expression level greater than 1.5 was required to qualify a gene as significantly differently expressed (Love et al., 2014). Significance for all other statistical analyses was set at *p* < 0.05.

## Results

**Figure 1:**
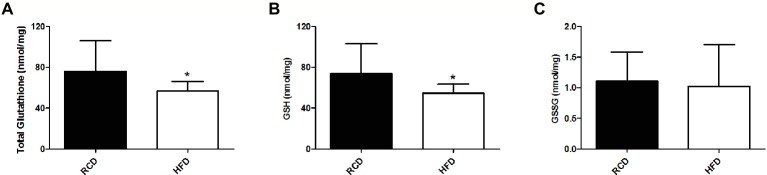
Soleus muscle levels of total glutathione **(A)**, GSH **(B)** and GSSG **(C)** in young rats submitted to 14 days of HFD or RCD. Results are presented as means ± SD for *n* = 12–13; * indicates significant difference between the two groups (*p* < 0.05).

**Figure 2:**
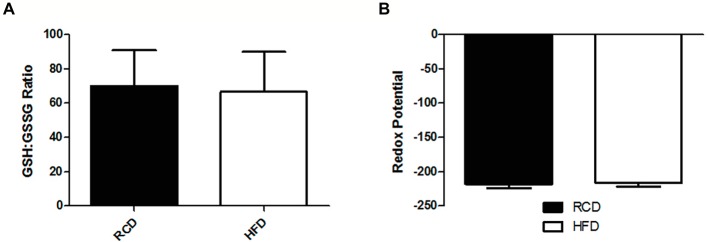
Soleus muscle GSH:GSSG ratio **(A)** and glutathione redox potential **(B)** in young rats submitted to 14 days of HFD or RCD. Results are presented as means ± SD for *n* = 12–13.

As previously reported (Andrich et al., 2018a,b), we found no significant difference in body weight between both groups (data not shown). We observed significantly lower total glutathione levels in the soleus muscle of the HFD group (*p* = 0.046; Figure 1A), which was largely due to the significant 25% decrease of GSH levels (*p* = 0.042; Figure 1B). However, we did not find any difference in GSSG levels (*p* = 0.722; Figure 1C), GSH:GSSG ratio (*p* = 0.693; Figure 2A), or glutathione redox potential (*p* = 0.534; Figure 2B).

**Figure 3:**
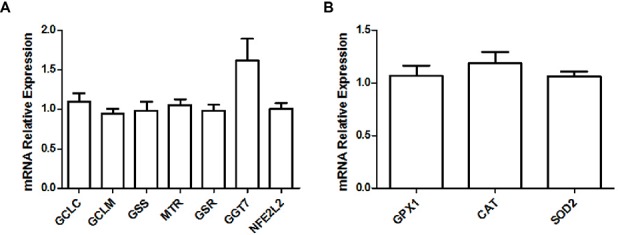
Relative gene expression levels of various enzymes and transcription factors implicated in the glutathione metabolism **(A)** and relative gene expression levels of major antioxidant enzymes **(B)** in the soleus muscle of young rats submitted to 14 days of HFD. Results are presented as mean fold change, compared to the RCD group, ±SEM for 5–6 replicates per condition.

When looking at gene expression levels, we did not find any significant differences in glutathione metabolism (Pizzorno, 2014) enzymes glutamate cysteine ligase catalytic subunit (GCLC; adjusted *p* = 0.865), glutamate cysteine ligase modifier subunit (GCLM; adjusted *p* = 0.800), glutathione synthase (GSS; adjusted *p* = 0.984), methionine synthase (MTR; adjusted *p* = 0.917), glutathione reductase (GSR; adjusted *p* = 0.978), γ-glutamyltransferase-7 (GGT7; adjusted *p* = 0.248), or the Nrf2 transcription factor (NFE2L2; adjusted *p* = 0.990; Figure 3A). Further, we did not find any differences in major antioxidant enzymes glutathione peroxidase (GPX; adjusted *p* = 0.912), catalase (CAT; adjusted *p* = 0.399), or mitochondrial superoxide dismutase (SOD2; adjusted *p* = 0.600; Figure 3B).

**Figure 4:**
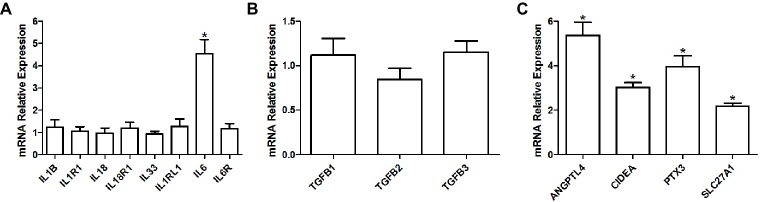
Relative gene expression levels of various interleukins and their respective receptors **(A)**, relative gene expression levels of TGF-β cytokines **(B)** as well as relative gene expression of various pro-inflammatory proteins **(C)** in the soleus muscle of young rats submitted to 14 days of HFD. Results are presented as mean fold change, compared to the RCD group, ±SEM for 5–6 replicates per condition; * indicates significant difference between the two groups (adjusted *p* < 0.10).

We observed a significant 4.5 fold increase in the expression of interleukin 6 (IL6; adjusted *p* = 0.05) in the HFD group, but not of its receptor (IL6R; adjusted *p* = 0.913) or of any other interleukins or their respective receptor (adjusted *p* ≥ 0.902; Figure 4A). Expression levels were also similar for the cytokine transforming growth factor β (TGFB; adjusted *p* ≥ 0.579; Figure 4B) superfamily genes. However, we observed a significant increase in the expression of other proteins implicated in pro-inflammatory pathways, such as a 5.4 fold increase in angiopoietin-like 4 (ANGPTL4; adjusted *p* = 0.009), a 3 fold increase in cell death activator CIDE-A (CIDEA; adjusted *p* < 0.001), a 4 fold increase in pentraxin-related protein PTX3 (PTX3; adjusted *p* = 0.006), and a 2.2 fold increase in long-chain fatty acid transport protein 1 (SLC27A1/FATP1; adjusted *p* < 0.001; Figure 4C).

**Figure 5:**
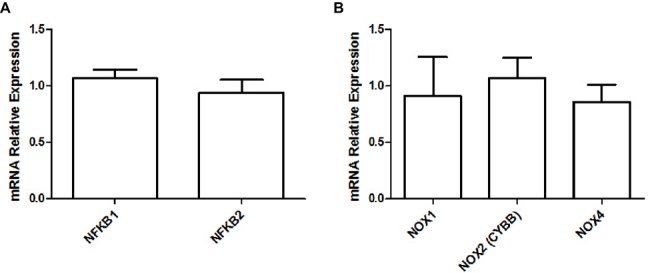
Relative gene expression levels of NF-κB **(A)** and relative gene expression levels of various NADPH oxidase isoforms **(B)** in the soleus muscle of young rats submitted to 14 days of HFD. Results are presented as mean fold change, compared to the RCD group, ±SEM for 5–6 replicates per condition.

Finally, we did not observe any difference in the gene expression levels of NF-κB (NFKB; adjusted *p* ≥ 0.801; Figure 5A) protein complex members or NADPH oxidase isoforms (NOX; adjusted *p* ≥ 0.801; Figure 5B) between both groups.

## Discussion

The present study intended to clarify the effects of a short-term HFD on the mechanisms regulating glutathione metabolism, the development of oxidative stress, and inflammation in the soleus (SOL) muscle of young rats. To our knowledge, the present results are first to demonstrate reduced glutathione levels after such a short exposure to HFD. After measuring the expression levels of a host of enzymes involved in the regulation of glutathione metabolism, results suggest that this decrease in GSH is not due to an alteration in *de novo* synthesis or GSSG recycling *via* GSR. Further, a significant increase in the expression of the pro-inflammatory cytokine interleukin 6 (IL-6) in the SOL muscle suggests an involvement in early metabolic alterations that can disrupt lipid and glucose metabolisms. These alterations precede any observable weight gain but could contribute to the mechanisms of impaired insulin signaling (Matsuzawa-Nagata et al., 2008), which could ultimately lead to the development of type 2 diabetes (Wang et al., 2003), as well as other metabolic disorders (Lumeng and Saltiel, 2011).

Multiple studies have previously reported HFD-induced altered GSH or GSSG levels or ratio in rodent skeletal muscle (Anderson et al., 2009; Ritchie and Dyck, 2012; Espinosa et al., 2013; Yuzefovych et al., 2013; Gortan Cappellari et al., 2016; Pinho et al., 2017). However, the expression of both GCL subunits (the rate-limiting enzymes in the *de novo* synthesis of GSH), or of GSR (catalyzing the reduction of GSSG into GSH), was not altered, as previously reported in mice liver (Zhou et al., 2018). The latter study hypothesized that HFD could alter GSH levels *via* glutathione synthesis-related gene promoters hypermethylation. In the same study, a diet supplemented with serine, a cysteine precursor, was shown to counteract the alterations in GSH production and the development of oxidative stress induced by a HFD in hepatic tissues. This is of great interest, since cysteine availability is the rate-limiting factor of cellular GSH synthesis (Lu, 2013). As shown in previous work (Andrich et al., 2018a), our HFD formulation is supplemented with L-cystine, the oxidized dimer form of cysteine. Therefore, we conclude that reduced glutathione levels observed in this study were not a consequence of decreased cysteine availability caused by a lack of nutritional intake, as L-cystine supplementation was previously shown to stimulate GSH production (Yin et al., 2016). In that same study, using the same diet and protocol, we observed significantly lighter livers in HFD rats (Andrich et al., 2018a). Glutathione levels are at their highest in liver (where it is primarily synthesized), which also plays an important role in glutathione inter-organ homeostasis (Ookhtens and Kaplowitz, 1998). However, it appears that hepatic GSH needs to reach extreme depletion before it can affect skeletal muscle GSH concentrations (Burk and Hill, 1995). Further, the hepatic cysteine concentration is not considered to be a limiting step of GSH synthesis, as methionine is converted to cysteine. Nevertheless, the first enzyme in this metabolic cascade, methionine adenosyltransferase, can be inhibited by oxidative molecules (Elremaly et al., 2012, 2016). On the other hand, a more recent study hypothesized that skeletal muscle glutamine levels could influence hepatic GSH production in the presence of oxidative stress (Bilinsky et al., 2015). Thus, HFD-modulated interactions between liver and skeletal muscle glutathione metabolism need to be clarified.

As GSH reduces hydrogen peroxide (H_2_O_2_) through GPX, the levels of GSSG, a product of that reaction, rise. Under cellular stress, GSH levels drop as GSSG accumulates in the cell, although it can also react with the free sulfhydryl group of a protein to form a mixed disulfide or be transported out of the cell (Lu, 2013). Hence, the GSH:GSSG ratio is a good indicator of cellular oxidative stress (Schafer and Buettner, 2001). We did not observe any significant HFD-induced changes in GSSG levels or in the GSH:GSSG ratio. This, combined with the lack of difference in the expression of major antioxidant enzymes GPX, CAT, and MnSOD or in various isoforms of NADPH oxidase, a superoxide precursor, would suggest that the soleus muscle is not under cellular stress, yet. This would confirm earlier findings from our group that showed no increase in ROS (H_2_O_2_) production from permeabilized soleus muscle fibers in rats submitted to the same 14-day HFD (Leduc-Gaudet et al., 2018). Nonetheless, *in vivo* measurements of H_2_O_2_ and malondialdehyde (MDA), a product of lipid peroxidation and widely used marker of oxidative stress (Nielsen et al., 1997), could provide further confirmation of these observations. The present results do not show any difference in the glutathione redox potential as calculated by the Nernst equation. However, this equation’s validity as an indicator of cellular redox potential is currently debated in the literature, as it appears that the redox potential is highly dependent of GSH but not GSSG levels (Flohe, 2013). Moreover, further evidence points toward the redox potential, depending predominantly on kinetics *per se* rather than thermodynamic constraints (Deponte, 2017). A major consequence of the observed 25% drop in GSH levels is a decreased capacity to detoxify endogenous peroxides *via* GPX. Thus, it is appealing to postulate that, under exercise-induced physical stress and accelerated ROS production (Steinbacher and Eckl, 2015), the SOL antioxidant defense system will be compromised in HFD rats due to significantly lower GSH levels. Measurements of skeletal muscle glutathione levels following an exercise bout could confirm this hypothesis.

**Figure 6:**
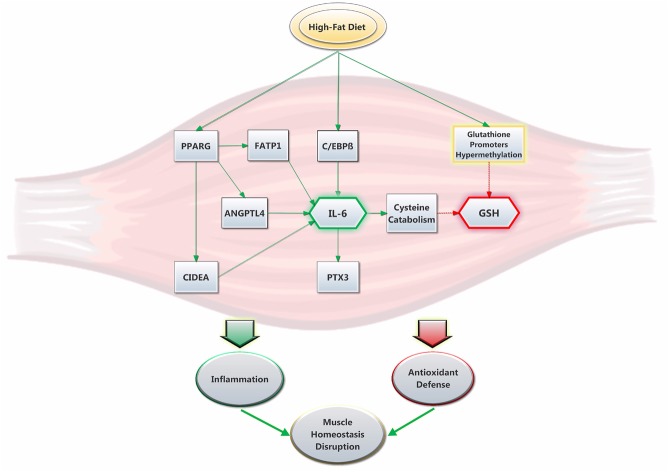
Suggested interplay between HFD, GSH levels and IL-6 expression in rat soleus muscle. The high-fat diet promptly promotes the expression of IL-6. This is stimulated by an increase in C/EBPβ (Hungness et al., 2002) and PPARγ activity (den Besten et al., 2015), the latter which yields the upregulation of pro-inflammatory proteins ANGPTL4, CIDEA and FATP1. In turn, IL-6 increases the expression of PTX3 and promotes cysteine catabolism (Hack et al., 1996), which lowers GSH levels. The latter are also decreased *via* HFD through a mechanism that was previously proposed to involve glutathione synthesis-related gene promoters hypermethylation (Zhou et al., 2018). Ultimately, the present results show that HFD promptly alters the antioxidant defense system while promoting inflammation and disruption in skeletal muscle homeostasis.

The other major finding of this study is the 4.5 fold increase in the gene expression of pro-inflammatory cytokine IL-6, which has previously been shown to be increased in the obese state (Eder et al., 2009) and diminished after weight loss (Bougoulia et al., 2006). Further, elevated IL-6 levels are a good indicator of an inflamed state, which can play a key role in the development of insulin resistance and other associated diseases (Yamashita et al., 2018). Its production can be regulated by various factors like C/EBPβ (Hungness et al., 2002) and PPARγ-activated proteins ANGPTL4 (Phua et al., 2017), CIDEA (Chatterjee et al., 2015), and FATP1 (Nishiyama et al., 2018). Interestingly, PPARγ has often been associated to Il-6 inhibition through STAT3 inactivation (Wang et al., 2004). However, other evidence suggests that PPARγ could trigger IL-6 production in skeletal muscle (Assi et al., 2017) and other cell types and tissues (Wanichkul et al., 2003; Zhang et al., 2014). Here, our data suggest that PPARγ, whose activity has been shown to be modulated by lipid ingestion (den Besten et al., 2015), could stimulate IL-6 expression through the activation of other proteins. In turn, IL-6 can induce the production of other pro-inflammatory proteins like PTX3 (Figure 6; Atar et al., 2017). Co-incidence of elevated IL-6 and lower GSH levels was previously reported (Valles et al., 2013), while obesogenic diets were shown to induce both of these effects in mice (Han et al., 2017) and rats (Govindaraj and Sorimuthu Pillai, 2015). In individuals with type 2 diabetes, increased IL-6 levels have been suspected as a cause of lowered GSH levels (Lagman et al., 2015). In mice, IL-6 was associated with glutathione depletion in the skeletal muscle, a mechanism possibly involving increased cysteine catabolism (Hack et al., 1996). Further, IL-6 has been shown to promote GSH release, but not production, from the liver into blood (Obrador et al., 2011). It remains to be seen if such a phenomenon could occur in the skeletal muscle. Furthermore, GSH has been shown to inhibit IL-6 production in patients with liver cirrhosis (Pena et al., 1999). Induced GSH depletion has also been demonstrated to inhibit T helper cell T_h_1 response in favor of T_h_2 response, which is responsible for IL-6 production (Peterson et al., 1998; Brundu et al., 2016). Thus, elevated IL-6 expression could also be consequential to low GSH levels. On the other hand, we did not observe a different expression of the IL-33 gene in the HFD group, which stimulates the production of T_h_2-associated cytokines (Schmitz et al., 2005). In both groups, we also found similar gene expression of other pro-inflammatory cytokines of the interleukin-1 superfamily, including IL-1β, whose expression has been shown to be stimulated by HFD in the *vastus lateralis* muscle of rats (Collins et al., 2016). In light of those results, it seems appropriate to recommend that the underlying mechanisms of glutathione and interleukin interactions should be further investigated in future studies.

In order to better assess the inflammatory status of the SOL muscle, the expression of TGF-β cytokine superfamily isoforms was also considered, as it was reported to decrease GSH levels in multiple cell types, *in vitro* (Liu and Gaston Pravia, 2010), possibly *via* the suppression of GCLC expression (Arsalane et al., 1997). Further, HFD was shown to induce a rise in TGF-β levels in both rats and mice (Yadav et al., 2011; Sousa-Pinto et al., 2016). We could not, however, observe similar results after submitting young rats to a 2-week HFD. Therefore, we cannot postulate that TGF-β influences glutathione metabolism at this early stage. As discussed above, a decrease in the GSH concentration, as a glutathione peroxidase substrate, will result in a lower detoxification of endogenous peroxide, allowing an increase in the intracellular concentration of H_2_O_2_. Because it activates NF-κB, H_2_O_2_ could therefore stimulate the increase of IL-6. Indeed, NF-κB is a major mediator of the inflammation cascade and its activation can be either stimulated or inhibited by GHS, depending on the tissue of interest and the experimental model (Hammond et al., 2001). Increased NF-κB phosphorylation was also observed in the gastrocnemius muscle of rats after 16 weeks of HFD (Sishi et al., 2011). Chronic NF-κB activation may be involved in the development of several diseases, including obesity and type 2 diabetes (Lira et al., 2012). We could not, however, detect any difference in the expression of NF-κB between HFD and RCD rats after 2 weeks, underlining that, in our model, HFD could impact endogenous metabolism rather than gene expression.

Results presented in this study demonstrate that a short-term high fat diet induces lower GSH levels in the SOL muscle of young rats. This effect can neither be attributed to a decrease in the expression of glutathione synthesis-implicated enzymes nor to observable oxidative stress. However, decreased GSH levels suggest a potentially altered antioxidant defense system. Moreover, 2 weeks of HFD induced a significant increase in IL-6 gene expression, which suggests its interaction with skeletal muscle glutathione metabolism. It was previously reported that IL-6 mRNA expression is increased in response to contractions or to glycogen depletion in the skeletal muscle (Munoz-Canoves et al., 2013). The present data, coinciding with our previous results, raise the hypothesis that disruptions in the antioxidant defense system, coupled to inflammation activation, could play a role in the impairment of contractile functions in the soleus muscle of young rats submitted to only 14 days of HFD. It was previously shown that IL-6 plays a pivotal role in muscle wasting mechanisms (Belizario et al., 2016), although we could not observe evidence of atrophy in neither SOL nor EDL in our previous results (Andrich et al., 2018b). On the other hand, GSH has been shown to improve Ca^2+^ sensitivity in rat skeletal muscle, although this was only observed in fast twitch fibers (Murphy et al., 2008) in which fast skeletal muscle troponin isoforms are highly expressed. Further, it appears as though diets rich in saturated fatty acids could alter fast skeletal muscle troponin T (TNNT3 gene) expression through alternative splicing of pre-mRNA in rat skeletal muscle (Black et al., 2017), although it remains to be seen if HFD could also alter the expression of other proteins of the troponin complex (troponin C and troponin I). Thus, further studies are needed to elucidate what role glutathione and inflammation could play in impaired oxidative muscle contractile functions and to better clarify the mechanisms (including the role of the liver and IL-6) underlying the reduction of GSH levels observed in the SOL muscle of young rats submitted to a short-term HFD.

## Ethics Statement

This study was carried out in strict accordance with recommendations of the National Institutes of Health guide for the care and use of Laboratory animals. Before undergoing the experimental work, the protocol was approved by the *Comité Institutionnel de Protection des Animaux* (CIPA) of UQAM (Permit Number: 0515-R3-759-0516).

## Author Contributions

DA and DS-P wrote the manuscript. DA, LM, YO, NA, JM, J-CG, LB, J-CL, and DS-P performed the experiments. DA contributed to data treatment, statistical analyses, tables and figures. FL, GD, AC, J-CL, and DS-P designed the study. All authors contributed to manuscript revision, read and approved the submitted version.

### Conflict of Interest Statement

The authors declare that the research was conducted in the absence of any commercial or financial relationships that could be construed as a potential conflict of interest.

## References

[ref1] Anderson E. J. Lustig M. E. Boyle K. E. Woodlief T. L. Kane D. A. Lin C. T.. (2009). Mitochondrial H_2_O_2_ emission and cellular redox state link excess fat intake to insulin resistance in both rodents and humans. J. Clin. Invest. 119, 573–581. 10.1172/JCI37048, PMID: 19188683 PMC2648700

[ref2] Andrich D. E. Melbouci L. Ou Y. Leduc-Gaudet J. P. Chabot F. Lalonde F. (2018a). Altered feeding behaviors and adiposity precede observable weight gain in young rats submitted to a short-term high-fat diet. J. Nutr. Metab. 2018. 10.1155/2018/1498150 PMC5901484 29805802

[ref3] Andrich D. E. Ou Y. Melbouci L. Leduc-Gaudet J. P. Auclair N. Mercier J. (2018b). Altered lipid metabolism impairs skeletal muscle force in young rats submitted to a short-term high-fat diet. Front. Physiol. 9:1327. 10.3389/fphys.2018.01327 30356919 PMC6190893

[ref4] Arsalane K. Dubois C. M. Muanza T. Begin R. Boudreau F. Asselin C.. (1997). Transforming growth factor-beta1 is a potent inhibitor of glutathione synthesis in the lung epithelial cell line A549: transcriptional effect on the GSH rate-limiting enzyme gamma-glutamylcysteine synthetase. Am. J. Respir. Cell Mol. Biol. 17, 599–607. 10.1165/ajrcmb.17.5.2833, PMID: 9374111

[ref5] Assi M. Kenawi M. Ropars M. Rebillard A. (2017). Interleukin-6, C/EBP-beta and PPAR-gamma expression correlates with intramuscular liposarcoma growth in mice: the impact of voluntary physical activity levels. Biochem. Biophys. Res. Commun. 490, 1026–1032. 10.1016/j.bbrc.2017.06.158, PMID: 28668397

[ref6] Atar A. Kural A. Yenice G. Comez I. Tugcu V. (2017). Role of interleukin-6 and pentraxin 3 as an early marker in Peyronie's disease. Kaohsiung J. Med. Sci. 33, 195–200. 10.1016/j.kjms.2017.01.007, PMID: 28359407 PMC11916746

[ref7] Auberval N. Dal S. Bietiger W. Pinget M. Jeandidier N. Maillard-Pedracini E.. (2014). Metabolic and oxidative stress markers in Wistar rats after 2 months on a high-fat diet. Diabetol. Metab. Syndr. 6:130. 10.1186/1758-5996-6-130, PMID: 25960774 PMC4424531

[ref8] Belizario J. E. Fontes-Oliveira C. C. Borges J. P. Kashiabara J. A. Vannier E. (2016). Skeletal muscle wasting and renewal: a pivotal role of myokine IL-6. Springerplus 5:619. 10.1186/s40064-016-2197-2 27330885 PMC4870483

[ref9] Bilinsky L. M. Reed M. C. Nijhout H. F. (2015). The role of skeletal muscle in liver glutathione metabolism during acetaminophen overdose. J. Theor. Biol. 376, 118–133. 10.1016/j.jtbi.2015.04.006, PMID: 25890031 PMC4431659

[ref10] Biro F. M. Wien M. (2010). Childhood obesity and adult morbidities. Am. J. Clin. Nutr. 91, 1499S–1505S. 10.3945/ajcn.2010.28701B, PMID: 20335542 PMC2854915

[ref11] Black A. J. Ravi S. Jefferson L. S. Kimball S. R. Schilder R. J. (2017). Dietary fat quantity and type induce transcriptome-wide effects on alternative splicing of pre-mRNA in rat skeletal muscle. J. Nutr. 147, 1648–1657. 10.3945/jn.117.254482, PMID: 28768832 PMC5572497

[ref12] Bougoulia M. Triantos A. Koliakos G. (2006). Plasma interleukin-6 levels, glutathione peroxidase and isoprostane in obese women before and after weight loss. Association with cardiovascular risk factors. Hormones 5, 192–199. 10.14310/horm.2002.11182, PMID: 16950753

[ref13] Brundu S. Palma L. Picceri G. G. Ligi D. Orlandi C. Galluzzi L.. (2016). Glutathione depletion is linked with Th2 polarization in mice with a retrovirus-induced immunodeficiency syndrome, murine AIDS: role of proglutathione molecules as immunotherapeutics. J. Virol. 90, 7118–7130. 10.1128/JVI.00603-16, PMID: 27226373 PMC4984623

[ref14] Burk R. F. Hill K. E. (1995). Reduced glutathione release into rat plasma by extrahepatic tissues. Am. J. Phys. 269, G396–G399. 10.1152/ajpgi.1995.269.3.G396, PMID: 7573450

[ref15] Charan J. Kantharia N. D. (2013). How to calculate sample size in animal studies? J. Pharmacol. Pharmacother. 4, 303–306. 10.4103/0976-500X.119726, PMID: 24250214 PMC3826013

[ref16] Chatterjee A. Mondal P. Ghosh S. Mehta V. S. Sen E. (2015). PPARgamma regulated CIDEA affects pro-apoptotic responses in glioblastoma. Cell Death Dis. 1:15038. 10.1038/cddiscovery.2015.38 PMC4979534 27551468

[ref17] Collins K. H. Hart D. A. Reimer R. A. Seerattan R. A. Waters-Banker C. Sibole S. C.. (2016). High-fat high-sucrose diet leads to dynamic structural and inflammatory alterations in the rat vastus lateralis muscle. J. Orthop. Res. 34, 2069–2078. 10.1002/jor.23230, PMID: 26990324

[ref18] DeFronzo R. A. Tripathy D. (2009). Skeletal muscle insulin resistance is the primary defect in type 2 diabetes. Diabetes Care 32(Suppl. 2), S157–S163. 10.2337/dc09-S302 19875544 PMC2811436

[ref19] den Besten G. Bleeker A. Gerding A. van Eunen K. Havinga R. van Dijk T. H.. (2015). Short-chain fatty acids protect against high-fat diet-induced obesity via a PPARgamma-dependent switch from lipogenesis to fat oxidation. Diabetes 64, 2398–2408. 10.2337/db14-1213, PMID: 25695945

[ref20] Deponte M. (2017). The incomplete glutathione puzzle: just guessing at numbers and figures? Antioxid. Redox Signal. 27, 1130–1161. 10.1089/ars.2017.7123, PMID: 28540740 PMC5661824

[ref21] Diaz-Morales N. Rovira-Llopis S. Escribano-Lopez I. Banuls C. Lopez-Domenech S. Falcon R.. (2016). Role of oxidative stress and mitochondrial dysfunction in skeletal muscle in type 2 diabetic patients. Curr. Pharm. Des. 22, 2650–2656. 10.2174/1381612822666160217142949, PMID: 26898744

[ref22] Eder K. Baffy N. Falus A. Fulop A. K. (2009). The major inflammatory mediator interleukin-6 and obesity. Inflamm. Res. 58, 727–736. 10.1007/s00011-009-0060-4, PMID: 19543691

[ref23] Elremaly W. Mohamed I. Rouleau T. Lavoie J. C. (2016). Impact of glutathione supplementation of parenteral nutrition on hepatic methionine adenosyltransferase activity. Redox Biol. 8, 18–23. 10.1016/j.redox.2015.12.003, PMID: 26722840 PMC4710792

[ref24] Elremaly W. Rouleau T. Lavoie J. C. (2012). Inhibition of hepatic methionine adenosyltransferase by peroxides contaminating parenteral nutrition leads to a lower level of glutathione in newborn Guinea pigs. Free Radic. Biol. Med. 53, 2250–2255. 10.1016/j.freeradbiomed.2012.10.541, PMID: 23085223

[ref25] Espinosa A. Campos C. Diaz-Vegas A. Galgani J. E. Juretic N. Osorio-Fuentealba C.. (2013). Insulin-dependent H_2_O_2_ production is higher in muscle fibers of mice fed with a high-fat diet. Int. J. Mol. Sci. 14, 15740–15754. 10.3390/ijms140815740, PMID: 23899788 PMC3759883

[ref26] Fisher-Wellman K. H. Gilliam L. A. Lin C. T. Cathey B. L. Lark D. S. Neufer P. D. (2013). Mitochondrial glutathione depletion reveals a novel role for the pyruvate dehydrogenase complex as a key H_2_O_2_-emitting source under conditions of nutrient overload. Free Radic. Biol. Med. 65, 1201–1208. 10.1016/j.freeradbiomed.2013.09.008, PMID: 24056031 PMC3965186

[ref27] Flohe L. (2013). The fairytale of the GSSG/GSH redox potential. Biochim. Biophys. Acta 1830, 3139–3142. 10.1016/j.bbagen.2012.10.020 23127894

[ref28] Frontera W. R. Ochala J. (2015). Skeletal muscle: a brief review of structure and function. Calcif. Tissue Int. 96, 183–195. 10.1007/s00223-014-9915-y, PMID: 25294644

[ref29] Galassetti P. (2012). Inflammation and oxidative stress in obesity, metabolic syndrome, and diabetes. Exp. Diabetes Res. 2012:943706. 10.1155/2012/943706, PMID: 23319940 PMC3540748

[ref30] Giacco F. Brownlee M. (2010). Oxidative stress and diabetic complications. Circ. Res. 107, 1058–1070. 10.1161/CIRCRESAHA.110.223545, PMID: 21030723 PMC2996922

[ref31] Gortan Cappellari G. Zanetti M. Semolic A. Vinci P. Ruozi G. Falcione A.. (2016). Unacylated ghrelin reduces skeletal muscle reactive oxygen species generation and inflammation and prevents high-fat diet-induced hyperglycemia and whole-body insulin resistance in rodents. Diabetes 65, 874–886. 10.2337/db15-1019, PMID: 26822085

[ref32] Govindaraj J. Sorimuthu Pillai S. (2015). Rosmarinic acid modulates the antioxidant status and protects pancreatic tissues from glucolipotoxicity mediated oxidative stress in high-fat diet: streptozotocin-induced diabetic rats. Mol. Cell. Biochem. 404, 143–159. 10.1007/s11010-015-2374-6, PMID: 25735949

[ref33] Hack V. Gross A. Kinscherf R. Bockstette M. Fiers W. Berke G. (1996). Abnormal glutathione and sulfate levels after interleukin 6 treatment and in tumor-induced cachexia. FASEB J. 10, 1219–1226. 8751725 10.1096/fasebj.10.10.8751725

[ref34] Hammond C. L. Lee T. K. Ballatori N. (2001). Novel roles for glutathione in gene expression, cell death, and membrane transport of organic solutes. J. Hepatol. 34, 946–954. 10.1016/S0168-8278(01)00037-X, PMID: 11451183

[ref35] Han H. Qiu F. Zhao H. Tang H. Li X. Shi D. (2017). Dietary flaxseed oil prevents western-type diet-induced nonalcoholic fatty liver disease in apolipoprotein-e knockout mice. Oxidative Med. Cell. Longev. 2017:3256241. 10.1155/2017/3256241, PMID: 29081885 PMC5610846

[ref36] Head G. A. (2015). Cardiovascular and metabolic consequences of obesity. Front. Physiol. 6:32. 10.3389/fphys.2015.00032, PMID: 25713539 PMC4322831

[ref37] Hruby A. Hu F. B. (2015). The epidemiology of obesity: a big picture. PharmacoEconomics 33, 673–689. 10.1007/s40273-014-0243-x, PMID: 25471927 PMC4859313

[ref38] Hungness E. S. Luo G. J. Pritts T. A. Sun X. Robb B. W. Hershko D.. (2002). Transcription factors C/EBP-beta and -delta regulate IL-6 production in IL-1beta-stimulated human enterocytes. J. Cell. Physiol. 192, 64–70. 10.1002/jcp.10116, PMID: 12115737

[ref39] Lagman M. Ly J. Saing T. Kaur Singh M. Vera Tudela E. Morris D.. (2015). Investigating the causes for decreased levels of glutathione in individuals with type II diabetes. PLoS One 10:e0118436. 10.1371/journal.pone.0118436, PMID: 25790445 PMC4366217

[ref40] Lavoie J. C. Rouleau T. Tsopmo A. Friel J. Chessex P. (2008). Influence of lung oxidant and antioxidant status on alveolarization: role of light-exposed total parenteral nutrition. Free Radic. Biol. Med. 45, 572–577. 10.1016/j.freeradbiomed.2008.04.018, PMID: 18474254

[ref41] Leduc-Gaudet J. P. Reynaud O. Chabot F. Mercier J. Andrich D. E. St-Pierre D. H.. (2018). The impact of a short-term high-fat diet on mitochondrial respiration, reactive oxygen species production, and dynamics in oxidative and glycolytic skeletal muscles of young rats. Physiol. Rep. 6. 10.14814/phy2.13548, PMID: 29479852 PMC6430054

[ref42] Lira F. S. Rosa J. C. Pimentel G. D. Seelaender M. Damaso A. R. Oyama L. M.. (2012). Both adiponectin and interleukin-10 inhibit LPS-induced activation of the NF-Î°B pathway in 3T3-L1 adipocytes. Cytokine 57, 98–106. 10.1016/j.cyto.2011.10.001, PMID: 22047972

[ref43] Liu R. M. Gaston Pravia K. A. (2010). Oxidative stress and glutathione in TGF-beta-mediated fibrogenesis. Free Radic. Biol. Med. 48, 1–15. 10.1016/j.freeradbiomed.2009.09.026, PMID: 19800967 PMC2818240

[ref44] Love M. I. Huber W. Anders S. (2014). Moderated estimation of fold change and dispersion for RNA-seq data with DESeq2. Genome Biol. 15:550. 10.1186/s13059-014-0550-8 25516281 PMC4302049

[ref45] Lu S. C. (2013). Glutathione synthesis. Biochim. Biophys. Acta 1830, 3143–3153. 10.1016/j.bbagen.2012.09.008 22995213 PMC3549305

[ref46] Lumeng C. N. Saltiel A. R. (2011). Inflammatory links between obesity and metabolic disease. J. Clin. Invest. 121, 2111–2117. 10.1172/JCI57132, PMID: 21633179 PMC3104776

[ref47] Matsuzawa-Nagata N. Takamura T. Ando H. Nakamura S. Kurita S. Misu H.. (2008). Increased oxidative stress precedes the onset of high-fat diet-induced insulin resistance and obesity. Metabolism 57, 1071–1077. 10.1016/j.metabol.2008.03.010, PMID: 18640384

[ref48] Munoz-Canoves P. Scheele C. Pedersen B. K. Serrano A. L. (2013). Interleukin-6 myokine signaling in skeletal muscle: a double-edged sword? FEBS J. 280, 4131–4148. 10.1111/febs.12338, PMID: 23663276 PMC4163639

[ref49] Murphy R. M. Dutka T. L. Lamb G. D. (2008). Hydroxyl radical and glutathione interactions alter calcium sensitivity and maximum force of the contractile apparatus in rat skeletal muscle fibres. J. Physiol. 586, 2203–2216. 10.1113/jphysiol.2007.150516, PMID: 18308823 PMC2465198

[ref50] Nielsen F. Mikkelsen B. B. Nielsen J. B. Andersen H. R. Grandjean P. (1997). Plasma malondialdehyde as biomarker for oxidative stress: reference interval and effects of life-style factors. Clin. Chem. 43, 1209–12140. PMID: 9216458

[ref51] Nishiyama K. Fujita T. Fujimoto Y. Nakajima H. Takeuchi T. Azuma Y. T. (2018). Fatty acid transport protein 1 enhances the macrophage inflammatory response by coupling with ceramide and c-Jun N-terminal kinase signaling. Int. Immunopharmacol. 55, 205–215. 10.1016/j.intimp.2017.12.003, PMID: 29272817

[ref52] Obrador E. Benlloch M. Pellicer J. A. Asensi M. Estrela J. M. (2011). Intertissue flow of glutathione (GSH) as a tumor growth-promoting mechanism: interleukin 6 induces GSH release from hepatocytes in metastatic B16 melanoma-bearing mice. J. Biol. Chem. 286, 15716–15727. 10.1074/jbc.M110.196261, PMID: 21393247 PMC3091180

[ref53] Ookhtens M. Kaplowitz N. (1998). Role of the liver in interorgan homeostasis of glutathione and cyst(e)ine. Semin. Liver Dis. 18, 313–329. 10.1055/s-2007-1007167 9875551

[ref54] Pai A. A. Baharian G. Page Sabourin A. Brinkworth J. F. Nedelec Y. Foley J. W.. (2016). Widespread shortening of 3' untranslated regions and increased exon inclusion are evolutionarily conserved features of innate immune responses to infection. PLoS Genet. 12:e1006338. 10.1371/journal.pgen.1006338, PMID: 27690314 PMC5045211

[ref55] Peckham S. C. Entenman C. Carroll H. W. (1962). The influence of a hypercalric diet on gross body and adipose tissue composition in the rat. J. Nutr. 77, 187–197. 10.1093/jn/77.2.187, PMID: 14484832

[ref56] Pena L. R. Hill D. B. McClain C. J. (1999). Treatment with glutathione precursor decreases cytokine activity. JPEN J. Parenter. Enteral Nutr. 23, 1–6. 10.1177/014860719902300101 9888410

[ref57] Peterson J. D. Herzenberg L. A. Vasquez K. Waltenbaugh C. (1998). Glutathione levels in antigen-presenting cells modulate Th1 versus Th2 response patterns. Proc. Natl. Acad. Sci. U. S. A. 95, 3071–3076. 9501217 10.1073/pnas.95.6.3071 PMC19696

[ref58] Phua T. Sng M. K. Tan E. H. Chee D. S. Li Y. Wee J. W. (2017). Angiopoietin-like 4 mediates colonic inflammation by regulating chemokine transcript stability via tristetraprolin. Sci. Rep. 7:44351. 10.1038/srep44351 28287161 PMC5347094

[ref59] Pinho R. A. Sepa-Kishi D. M. Bikopoulos G. Wu M. V. Uthayakumar A. Mohasses A.. (2017). High-fat diet induces skeletal muscle oxidative stress in a fiber type-dependent manner in rats. Free Radic. Biol. Med. 110, 381–389. 10.1016/j.freeradbiomed.2017.07.005, PMID: 28690197

[ref60] Pizzorno J. (2014). Glutathione! Integr. Med. 13, 8–12. PMC4684116 26770075

[ref61] Rao D. P. Kropac E. Do M. T. Roberts K. C. Jayaraman G. C. (2016). Childhood overweight and obesity trends in Canada. Health Promot. Chronic Dis. Prev. Can. 36, 194–198. 10.24095/hpcdp.36.9.03, PMID: 27670922 PMC5129778

[ref62] Ritchie I. R. Dyck D. J. (2012). Rapid loss of adiponectin-stimulated fatty acid oxidation in skeletal muscle of rats fed a high fat diet is not due to altered muscle redox state. PLoS One 7:e52193. 10.1371/journal.pone.0052193, PMID: 23284930 PMC3524092

[ref63] Schafer F. Q. Buettner G. R. (2001). Redox environment of the cell as viewed through the redox state of the glutathione disulfide/glutathione couple. Free Radic. Biol. Med. 30, 1191–1212. 10.1016/S0891-5849(01)00480-4, PMID: 11368918

[ref64] Schmitz J. Owyang A. Oldham E. Song Y. Murphy E. McClanahan T. K.. (2005). IL-33, an interleukin-1-like cytokine that signals via the IL-1 receptor-related protein ST2 and induces T helper type 2-associated cytokines. Immunity 23, 479–490. 10.1016/j.immuni.2005.09.015, PMID: 16286016

[ref65] Sishi B. Loos B. Ellis B. Smith W. du Toit E. F. Engelbrecht A. M. (2011). Diet-induced obesity alters signalling pathways and induces atrophy and apoptosis in skeletal muscle in a prediabetic rat model. Exp. Physiol. 96, 179–193. 10.1113/expphysiol.2010.054189, PMID: 20952489

[ref66] Sousa-Pinto B. Goncalves L. Rodrigues A. R. Tomada I. Almeida H. Neves D.. (2016). Characterization of TGF-beta expression and signaling profile in the adipose tissue of rats fed with high-fat and energy-restricted diets. J. Nutr. Biochem. 38, 107–115. 10.1016/j.jnutbio.2016.07.017, PMID: 27736730

[ref67] Steinbacher P. Eckl P. (2015). Impact of oxidative stress on exercising skeletal muscle. Biomolecules 5, 356–377. 10.3390/biom5020356, PMID: 25866921 PMC4496677

[ref68] The N. S. Suchindran C. North K. E. Popkin B. M. Gordon-Larsen P. (2010). Association of adolescent obesity with risk of severe obesity in adulthood. JAMA 304, 2042–2047. 10.1001/jama.2010.1635, PMID: 21063014 PMC3076068

[ref69] Tsutsui H. Kinugawa S. Matsushima S. Yokota T. (2011). Oxidative stress in cardiac and skeletal muscle dysfunction associated with diabetes mellitus. J. Clin. Biochem. Nutr. 48, 68–71. 10.3164/jcbn.11-012FR, PMID: 21297915 PMC3022067

[ref70] Turcot V. Rouleau T. Tsopmo A. Germain N. Potvin L. Nuyt A. M.. (2009). Long-term impact of an antioxidant-deficient neonatal diet on lipid and glucose metabolism. Free Radic. Biol. Med. 47, 275–282. 10.1016/j.freeradbiomed.2009.04.026, PMID: 19409486

[ref71] Valles S. L. Benlloch M. Rodriguez M. L. Mena S. Pellicer J. A. Asensi M.. (2013). Stress hormones promote growth of B16-F10 melanoma metastases: an interleukin 6- and glutathione-dependent mechanism. J. Transl. Med. 11:72. 10.1186/1479-5876-11-72, PMID: 23517603 PMC3608962

[ref72] Vial G. Dubouchaud H. Couturier K. Cottet-Rousselle C. Taleux N. Athias A.. (2011). Effects of a high-fat diet on energy metabolism and ROS production in rat liver. J. Hepatol. 54, 348–356. 10.1016/j.jhep.2010.06.044, PMID: 21109325

[ref73] Wang X. Feng Z. Yang L. Han S. Cao K. Xu J.. (2016). O-GlcNAcase deficiency suppresses skeletal myogenesis and insulin sensitivity in mice through the modulation of mitochondrial homeostasis. Diabetologia 59, 1287–1296. 10.1007/s00125-016-3919-2, PMID: 26993634

[ref74] Wang Y. Wang P. Y. Qin L. Q. Davaasambuu G. Kaneko T. Xu J.. (2003). The development of diabetes mellitus in Wistar rats kept on a high-fat/low-carbohydrate diet for long periods. Endocrine 22, 85–92. 10.1385/ENDO:22:2:85, PMID: 14665711

[ref75] Wang L. H. Yang X. Y. Zhang X. Huang J. Hou J. Li J.. (2004). Transcriptional inactivation of STAT3 by PPARgamma suppresses IL-6-responsive multiple myeloma cells. Immunity 20, 205–218. 10.1016/S1074-7613(04)00030-5, PMID: 14975242

[ref76] Wanichkul T. Han S. Huang R. P. Sidell N. (2003). Cytokine regulation by peroxisome proliferator-activated receptor gamma in human endometrial cells. Fertil. Steril. 79(Suppl. 1), 763–769. 10.1016/s0015-0282(02)04835-5 12620489

[ref77] Yadav H. Quijano C. Kamaraju A. K. Gavrilova O. Malek R. Chen W.. (2011). Protection from obesity and diabetes by blockade of TGF-beta/Smad3 signaling. Cell Metab. 14, 67–79. 10.1016/j.cmet.2011.04.013, PMID: 21723505 PMC3169298

[ref78] Yamashita A. S. Belchior T. Lira F. Bishop N. C. Wessner B. Rosa J. C. (2018). Regulation of metabolic disease-associated inflammation by nutrient sensors. Mediat. Inflamm. 2018:18. 10.1155/2018/8261432 PMC6079375 30116154

[ref79] Yin J. Ren W. Yang G. Duan J. Huang X. Fang R. (2016). L-Cysteine metabolism and its nutritional implications. Mol. Nutr. Food Res. 60, 134–146. 10.1002/mnfr.201500031 25929483

[ref80] Yokota T. Kinugawa S. Hirabayashi K. Matsushima S. Inoue N. Ohta Y.. (2009). Oxidative stress in skeletal muscle impairs mitochondrial respiration and limits exercise capacity in type 2 diabetic mice. Am. J. Physiol. Heart Circ. Physiol. 297, H1069–H1077. 10.1152/ajpheart.00267.2009, PMID: 19617406

[ref81] Yuzefovych L. V. Musiyenko S. I. Wilson G. L. Rachek L. I. (2013). Mitochondrial DNA damage and dysfunction, and oxidative stress are associated with endoplasmic reticulum stress, protein degradation and apoptosis in high fat diet-induced insulin resistance mice. PLoS One 8:e54059. 10.1371/journal.pone.0054059, PMID: 23342074 PMC3546973

[ref82] Zhang Y. Hu L. Cui Y. Qi Z. Huang X. Cai L.. (2014). Roles of PPARgamma/NF-kappaB signaling pathway in the pathogenesis of intrahepatic cholestasis of pregnancy. PLoS One 9:e87343. 10.1371/journal.pone.0087343, PMID: 24489901 PMC3906154

[ref83] Zhou X. He L. Zuo S. Zhang Y. Wan D. Long C. (2018). Serine prevented high-fat diet-induced oxidative stress by activating AMPK and epigenetically modulating the expression of glutathione synthesis-related genes. Biochim. Biophys. Acta 1864, 488–498. 10.1016/j.bbadis.2017.11.009 29158183

